# Potential Hematopoietic Effects of SGLT2 Inhibitors in Patients with Cardiac Amyloidosis

**DOI:** 10.31083/RCM26081

**Published:** 2025-03-20

**Authors:** Nikita Ermolaev, Robin Willixhofer, Christoph Krall, Christina Kronberger, René Rettl, Christina Binder, Franz Duca, Christian Nitsche, Andreas Kammerlander, Michael Poledniczek, Bernhard Gregshammer, Diana Ahmadi-Fazel, Mahshid Eslami, Luciana Camuz Ligios, Johannes Kastner, Jutta Bergler-Klein, Roza Badr Eslam

**Affiliations:** ^1^Division of Cardiology, Department of Internal Medicine II, Medical University of Vienna, 1090 Vienna, Austria; ^2^Center for Medical Data Science, Medical University of Vienna, 1090 Vienna, Austria

**Keywords:** amyloid cardiomyopathy, heart failure, sodium-glucose cotransporter 2 inhibitors, hematopoiesis, functional capacity, cardiopulmonary exercise testing

## Abstract

**Background:**

Sodium–glucose cotransporter 2 inhibitors (SGLT2i) have been found to have potential hematopoietic effects in patients with heart failure (HF). However, these benefits have not been studied in patients with cardiac amyloidosis (CA). CA patients present with HF symptoms and often suffer from iron deficiency, which has a negative impact on erythropoiesis and leads to lower hemoglobin and hematocrit levels. We sought to determine the potential effects of SGLT2i on hematological parameters and functional capacity (FC) in CA patients.

**Methods:**

A prospective analysis was conducted to compare the effects of SGLT2i in patients who received the best medical therapy (BMT) along with SGLT2i (n = 20), versus patients receiving only BMT without SGLT2i (n = 20) (historical control group). All patients underwent blood testing and cardiopulmonary exercise testing (CPET) at baseline (BL) and after 6 months [interquartile range (IQR): 4.0 to 8.0].

**Results:**

The SGLT2i-based therapy resulted in a significant improvement and difference in hematological parameters at 6 months follow-up compared to the control group. In the SGLT2i group, the mean hemoglobin level increased (+1.2 mg/dL), whereas in the control group, it decreased (–0.8 g/dL) (*p* < 0.001 for overall group comparison). The hematocrit showed a significant increase in the SGLT2i group (+4.4%) compared to a decrease in the control group (–1.8%) (*p* < 0.001). Additionally, the serum iron level improved in the SGLT2i-treated group (+ 5.5 [–5.0 to 17.5] μg/dL vs. –6.0 [–15.0 to 4.0] μg/dL, *p* = 0.121). Although there was no significant change in the peak oxygen consumption (peak VO_2_, (mL/min)/kg) (*p* = 0.206), as well as in pulmonary ventilation (VE)/carbon dioxide production (VCO_2_) slope in both groups (*p* = 0.964), the SGLT2i group maintained a peak VO_2_ and VE/VCO_2_ slope throughout the study.

**Conclusions:**

SGLT2i therapy improved hematological parameters and stabilized the FC of CA patients.

## 1. Introduction

Cardiac amyloidosis (CA) is a rare systemic, but serious condition characterized by the deposition of misfolded proteins (amyloid fibrils) in the myocardium [1]. The two most abundant amyloid fibrils, mainly infiltrating the heart are derived from immunoglobulin light chain amyloid (AL) and transthyretin amyloid (ATTR), both leading to restrictive cardiomyopathy (CM) [2]. In addition, ATTR differs between a non-hereditary wild-type (*ATTRwt*) and a variant form (*ATTRv*) [3].

CA typically tends to remain underdiagnosed or diagnosed in progressing disease stages, however, recent advances in diagnosis with non-invasive methods, as well as heightened disease recognition, have improved early diagnosis and management of patients with CA [4, 5, 6]. The amyloid fibril infiltration disrupts the normal function of the heart, which leads to various complications including heart failure (HF), primarily with preserved ejection fraction (HFpEF) [7, 8]. In addition, the presence of HF phenotypes contributes to lower iron levels and anemia, which is common in HF patients, independent of the etiology of HF. Anemia in HF leads to a lowcardiac output, an increased rate of hospitalizations and a low functional capacity (FC) [9, 10, 11]. Previous studies have shown that anemia leads to impaired FC as measured by cardiopulmonary exercise testing (CPET), which further contributes to poor patient outcomes [12, 13]

The current treatment options for CA include disease specific therapy (DST) with tafamidis, which has been found to be safe and effective in previous studies [14, 15, 16]. It is very important to treat concomitant conditions and the consequences of the underlying main disease with the best medical therapy (BMT). BMT of CA includes a multidisciplinary approach and focuses on symptomatic relief, management of conduction disorders and supportive care [6, 17].

Another safe and effective agent recently added to the management of HF, irrespective of the left ventricular ejection fraction (LVEF) are sodium-glucose cotransporter 2 inhibitors (SGLT2i) [18, 19, 20, 21, 22]. These therapeutic agents also have potential hematopoetic effects, and have been shown to increase hemoglobin levels in patients receiving these agents [23, 24, 25].

Previous study has shown that the emergence of SGLT2i is a promising avenue for the treatment for CA [26]. Despite the fact that SGLT2i in patients with CA were well-tolerated, its efficacy and safety in these patients are still underinverstigated, and no prior studies have directly explored the hematopoietic effects of SGLT2i specifically within the CA patient population [27, 28, 29]. This study seeks to address this gap, evaluating both the hematopoietic impact and the potential clinical benefits of SGLT2i in CA.

In fact, we hypothesized that treatment with SGLT2i might improve the hematopoietic effects in this collective. Therefore, we sought to investigate the contribution of SGLT2i in the management of CA, with a specific focus on their effects on hemoglobin and hematocrit levels and their impact on FC.

## 2. Methods

### 2.1 Study Design and Participants

We prospectively collected data on 40 patients with an average follow-up period of 6 months [interquartile range (IQR): 4.0 to 8.0], who had been diagnosed with CA in accordance with proposed diagnostic pathways [30] and received SGLT2i (either empagliflozin or dapagliflozin in therapeutic dose) in addition to BMT and DST at our center (n = 20) compared with a historical control group without SGLT2i (n = 20, only BMT and DST). SGLT2i therapy was indicated in stable amyloidosis patients as part of the management for heart failure. The historical control group was chosen from those patients who rejected the SGLT2i therapy or this therapy was discontinued by the primary care physician. The inclusion criteria were (1) confirmed diagnosis of cardiac amyloidosis; (2) treatment with tafamidis and individual BMT prior to baseline (BL) assessment; (3) ability to undergo CPET and (4) hemoglobin concentration less than or equal to 13.5 g/dL. Exclusion criteria were (1) presence of acute or chronic bleeding and (2) any documented history of bleeding disorders.

### 2.2 Diagnosis of Cardiac Amyloidosis

Patients were diagnosed in accordance with the proposed diagnostic pathways previously reported by Kittleson M *et al*. [30]. Although bone scintigraphy is considered as a gold standard of non-invasive ATTR-CM diagnosis [4], cardiac uptake that is consistent with ATTR-CM (grade 2 or 3 uptake) may be present in over 10% of patients with AL-CM [31]. Therefore, aside from bone scintigraphy, all patients underwent mandatory screening for paraprotein and monoclonal protein including 3 laboratory tests: serum free light chain (sFLC) assay, as well as serum and urine immunofixation. Gene sequencing was performed if patients had given written consent for genetic analysis.

### 2.3 Assessment of Laboratory Parameters

Laboratory analysis focused on the evaluation of parameters such as hemoglobin, hematocrit, mean corpuscular volume (MCV), mean corpuscular hemoglobin concentration (MCHC), red cell distribution width (RDW), as well as iron status, ferritin, and transferrin saturation (TSAT). Additionally, we assessed the cardiac marker N-terminal prohormone of brain natriuretic peptide (NT-proBNP) and kidney function using an estimated glomerular filtration rate (eGFR) calculated via the Modification of Diet in Renal Disease (MDRD) formula.

### 2.4 Evaluation of Functional Capacity

The 6-minute walking test (6MWT) was used as a sub-maximal measure to evaluate the patient’s functional status. CPET was conducted to objectively evaluate the cardiorespiratory system [32]. Patients performed symptom limited maximum CPET at BL and follow-up assessment (FUP) visits. CPET was performed using a cycle ergometer (Ergometer E Bike REF 2017911-007, GE Healthcare, Wauwatosa, WI, USA) with an incremental step protocol characterized by a gradual stepwise increase of work rate at each minute of exercise with a goal of ten minutes. Step protocols were individually chosen (intensity related to subjective daily physical activity and results of the 6MWT at BL).

Gas exchange parameters were collected using a face mask, with each parameter recorded breath-by-breath (Dual-Monitor Vyntus CPX SN 42600071, Carl Reiner, Austria). Vital parameters (electrocardiogram and heart rate) were continuously collected, with blood pressure measurements every two minutes (using GE CAM USB CardioSoft 12-channel-PC-ECG (GE Healthcare, Wauwatosa, WI, USA)). All parameters were assessed for up to two minutes at rest, during exercise and up to three minutes at recovery. Analyzed CPET variables included peak oxygen consumption (peak VO_2_), oxygen consumption (VO_2_) at anaerobic threshold (AT), peak pulmonary ventilation (peak VE) as well as pulmonary ventilation (VE)/(carbon dioxide production) VCO_2_ slope. Peak VO_2_ was defined as the highest 30-second value reached and identified by the disproportionate rise in VE relative to VO_2_. The AT was determined using the V-slope method and validated by ventilatory equivalent and end-tidal methods [33]. The VE/VCO_2_ slope was calculated as the slope of the linear relationship between VE and VCO_2_ after the beginning of loaded exercise to the end of the isocapnic buffering period [34]. Peak VE was calculated by multiplying the respiratory rate by the volume of air exhaled during each breathing cycle (tidal volume). The respiratory exchange ratio was determined as VCO_2_ divided by VO_2_.

### 2.5 Transthoracic Echocardiography

Transthoracic echocardiography (TTE) was conducted by certified professionals using state-of-the-art equipment (GE Vivid E95, Vivid E9, and Vivid 7, GE Healthcare, Wauwatosa, WI, USA) following current guidelines [35, 36]. Image interpretation was carried out after the assessment on a contemporary offline clinical workstation equipped with specialized software (Version 204, EchoPAC, GE Healthcare, Wauwatosa, WI, USA) by certified cardiologists.

### 2.6 Statistical Analysis

All statistical analysis were performed with R 4.4.0 (R Foundation, Vienna, Austria) and IBM SPSS Version 29.0 (IBM SPSS, Armonk, NY, USA). Continuous variables were reported as mean and standard deviation or as a median and IQR. Discrete variables are presented as percentage and numbers. Continuous data were compared using a *t*-test or Mann-Whitney U test and a chi-square test was used for categorical data for comparison between two groups. We performed univariate linear regression analysis for changes in laboratory and CPET parameters. For all tests, the two-tailed significance level was set at *p* < 0.05. No adaptation of the *p*-values for multiple hypothesis testing was performed.

## 3. Results

### 3.1 Demographic Data

Data from the 40 patients diagnosed with ATTR-CM patients were analyzed prior to and after treatment with SGLT2i (6.0 months, IQR: 4.0 to 8.0). 34 patients had a wild-type phenotype (85.0%), 4 patients had a variant form (10.0%), and 2 patients were diagnosed with a mixed phenotype (5.0%) of ATTR and AL-CM. Both patients with mixed phenotype underwent myocardial biopsy to evaluate the severity of amyloid fibril deposition. Immunohistochemical staining showed positive results for antibodies against ATTR, while staining for serum amyloid A (SAA) and light chains (IgG Kappa, IgG Lambda) was not significant. Additionally, both patients were diagnosed with Smoldering Myeloma and have not received specific treatment, as the disease has not met the CRAB (hypercalcaemia, renal failure, anaemia, bone lesions) criteria for progression [37]. Additionally, 78.0% of the participants were male. The average age at study entry was 78.5 (±6.4) years with significant difference between the groups (80.8 [±4.7] in patients without SGLT2i vs. 76.2 [±7.1] years in patients with SGLT2i therapy, *p* = 0.023).

Concerning the specific diagnosis of ATTR-CM, 37 patients had a Perugini grade of either 2 (27.5%) or (65.0%) and 3 patients (7.5%) had a positive myocardial biopsy.

With respect to comorbidities, no significant differences were observed between the groups, except for kidney function, which was slightly worse in the group not receiving SGLT2i therapy (*p* = 0.061). In the entire patient cohort, 11 (28.0%) had an intracardiac device (pacemaker or implanted cardioverter defibrillator), implanted before the start of the study.

Patients had no statistically significant differences in New York Heart Association (NYHA) functional class (NYHA ≥II 85.0% vs. 70.0%, *p* = 0.524), mean 6MWT distance (367.2 (±99.3) vs. 405.4 (±97.4) meters, *p* = 0.253), or median NT-proBNP levels (3773.5 [IQR: 1670.5 to 5224.5] vs. 2127.0 [IQR: 1117.2 to 3579.2], *p* = 0.097). Tables 1,2,3 provide further data on demographic characteristics, comorbidities, concomitant medication, as well as laboratory, TTE and CPET parameters at BL. There were no interruptions in SGLT2i therapy.

**Table 1.** **Patients population characteristics: clinical parameters, comorbidities, and concomitant medication**.

Variables		All	Patients without SGLT2i	Patients with SGLT2i	*p*-value
		(n = 40)	(n = 20)	(n = 20)	
Age, years		78.5 (6.4)	80.8 (4.7)	76.2 (7.1)	0.023
Sex, male		31 (78.0)	17 (85.0)	14 (70.0)	0.449
Body mass index, kg/m^2^		25.6 (3.1)	25.1 (2.3)	26.1 (3.7)	0.317
NYHA functional class					0.524
	Class I	9 (22.0)	3 (15.0)	6 (30.0)	
	Class II	20 (50.0)	11 (55.0)	9 (45.0)	
	Class III	11 (28.0)	6 (30.0)	5 (25.0)	
History of HF-hospitalization		3 (7.5)	1 (5.0)	2 (10.0)	0.553
6–minute walk distance, m		386.3 (98.9)	367.2 (99.3)	405.4 (97.4)	
*TTR *genotype					0.223
	*ATTRwt*	34 (85.0)	17 (85.0)	17 (85.0)	
	*ATTRv*	4 (10.0)	1 (5.0)	3 (15.0)	
	Mixed phenotype (*ATTRwt*+AL)	2 (5.0)	2 (10.0)	0 (0.0)	
Perugini Grading Scale (Grading)					0.357
	2	11 (27.5)	5 (25.0)	6 (30.0)	
	3	26 (65.0)	13 (65.0)	13 (65.0)	
Endomyocardial biopsy		3 (7.5)	2 (10.0)	1 (5.0)	
Comorbidities					
	Arterial hypertension	27 (68.0)	12 (60.0)	15 (75.0)	0.499
	Atrial fibrillation or flutter	22 (56.0)	11 (55.0)	11 (58.0)	1.000
	Coronary artery disease	11 (28.0)	5 (25.0)	6 (30.0)	1.000
	Hyperlipidemia	23 (58.0)	10 (50.0)	13 (65.0)	0.522
	Chronic kidney disease	18 (45.0)	12 (60.0)	6 (30.0)	0.061
	Diabetes mellitus II	6 (15.0)	3 (15.0)	3 (15.0)	1.000
	COPD	7 (18.0)	6 (30.0)	1 (5.0)	0.096
	Smoker	1 (3.0)	1 (5.0)	0 (0.0)	0.979
	Polyneuropathy	16 (44.0)	7 (35.0)	9 (45.0)	1.000
Concomitant medication					
	Anticoagulants	24 (60.0)	13 (65.0)	11 (55.0)	0.747
	Antiplatelets	10 (25.0)	7 (35.0)	3 (15.0)	0.273
	Beta-blockers	24 (60.0)	11 (55.0)	13 (65.0)	0.747
	ACE-I/ARBs/ARNI	18 (45.0)	8 (40.0)	10 (50.0)	0.751
	MRA	19 (48.0)	13 (65.0)	6 (30.0)	0.058
	Diuretics	27 (68.0)	13 (65.0)	14 (70.0)	1.000
	Lipid-lowering drugs	23 (58.0)	11 (55.0)	12 (60.0)	1.000
	Intracardiac device, yes	11 (28.0)	4 (20.0)	7 (35.0)	0.479

This table describes the patient baseline characteristics. Values are a mean ± SD or N (%). ACE-I, angiotensin-converting enzyme inhibitor; AL, immunoglobulin light chain amyloid; ARBs, angiotensin II receptor blockers; ARNI, angiotensin receptor neprilysin inhibitors; *ATTRv*, variant transthyretin amyloid; *ATTRwt*, wild-type transthyretin amyloid; COPD, chronic obstructive pulmonary disease; MRA, mineralocorticoid receptor antagonist; NYHA, New York Heart Association; TTR, transthyretin protein; SGLT2i, sodium–glucose cotransporter 2 inhibitors; HF, heart failure.

**Table 2.** **Patients population characteristics: laboratory and echocardiographic parameters**.

Variables	All (n = 40)	Patients without SGLT2i (n = 20)	Patients with SGLT2i (n = 20)	*p*-value
Hemoglobin, g/dL	12.8 (11.9 to 13.1)	12.5 (11.7 to 13.0)	12.9 (12.6 to 13.1)	0.239
Hematocrit, %	37.8 (35.6 to 39.4)	36.8 (35.5 to 38.1)	39.2 (37.4 to 39.6)	0.050
MCV, fL	92.8 (88.3 to 95.3)	92.8 (88.9 to 95.5)	92.6 (87.8 to 95.0)	0.089
MCHC, g/dL	33.5 (32.5 to 34.0)	33.7 (32.5 to 34.2)	33.0 (32.6 to 33.8)	0.291
RDW, %	14.1 (13.3 to 15.1)	14.2 (13.6 to 15.3)	13.9 (12.7 to 15.0)	0.189
Iron blood level, µg/dL	65.0 (50.5 to 79.5)	61.0 (48.0 to 76.5)	70.5 (61.0 to 78.8)	0.312
Serum ferritin, µg/dL	130.9 (77.4 to 222.0)	134.6 (75.8 to 254.7)	130.9 (91.2 to 219.2)	0.915
TSAT, %	17.8 (13.6 to 23.9)	17.0 (11.7 to 22.0)	21.4 (15.1 to 24.1)	0.247
NT-proBNP, pg/mL	2583.0 (1278.0 to 4725.0)	3773.5 (1670.0 to 5224.0)	2127.0 (1117.0 to 3579.0)	0.097
eGFR, mL/min/1.73 m^2^	49.8 (37.2 to 76.8)	42.6 (33.7 to 60.0)	67.6 (48.3 to 81.8)	<0.050
CRP, mg/L	0.2 (0.1 to 0.4)	0.2 (0.1 to 0.4)	0.2 (0.1 to 0.3)	0.380
HFrEF/HFmrEF/HFpEF, %	(17.5; 60.0; 17.5)	(20.0; 30.0; 50.0)	(15.0; 5.0; 70.0)	0.095

Values are a median and interquartile range [IQR] or N (%). CRP, C-reactive protein; eGFR, estimated glomerular filtration rate; HFmrEF, heart failure with mild reduced ejection fraction; HFpEF, heart failure with preserved ejection fraction; HFrEF, heart failure with reduced ejection fraction; MCHC, mean corpuscular hemoglobin concentration; MCV, mean corpuscular volume; NT-proBNP, N-terminal prohormone of brain natriuretic peptide; RDW, red cell distribution width; TSAT, transferrin saturation; SGLT2i, sodium–glucose cotransporter 2 inhibitors.

**Table 3.** **Patients population characteristics: cardiopulmonary exercise test parameters**.

Variables	All (n = 40)	Patients without SGLT2i (n = 20)	Patients with SGLT2i (n = 20)	*p*-value
Peak VO_2_, mL/min	1014 (921 to 1271)	982 (863 to 1200)	1032 (942 to 1460)	0.248
Peak VO_2_, mL/min/kg	14 (12 to 18)	13 (12 to 16)	14 (12 to 18)	0.637
VO_2_ at AT, mL/min	8 (7 to 10)	7 (6 to 7)	8 (7 to 9)	0.002
VE/VCO_2_ slope	38 (33 to 43)	38 (33 to 42)	38 (33 to 44)	1.000
Peak VE, L/min	52 (43 to 62)	48 (42 to 58)	53 (47 to 67)	0.101
Peak workload, Watt	70 (56 to 90)	56 (55 to 90)	77.0 (60 to 90)	0.377
Peak RER	1.1 (1 to 2)	1.1 (1 to 2)	1.1 (1 to 2)	0.704
Peak HR, bpm	116 (110 to 131)	115 (110 to 129)	118 (112 to 133)	0.579

Values are a median and interquartile rage [IQR]. AT, anaerobic threshold; HR, heart ratio; RER, respiratory exchange ratio; VE, pulmonary ventilation; VO_2_, oxygen consumption; SGLT2i, sodium–glucose cotransporter 2 inhibitors; VCO_2_, carbon dioxide production.

### 3.2 Changes in Laboratory and Echocardiographic Parameters

**Fig. 1.**
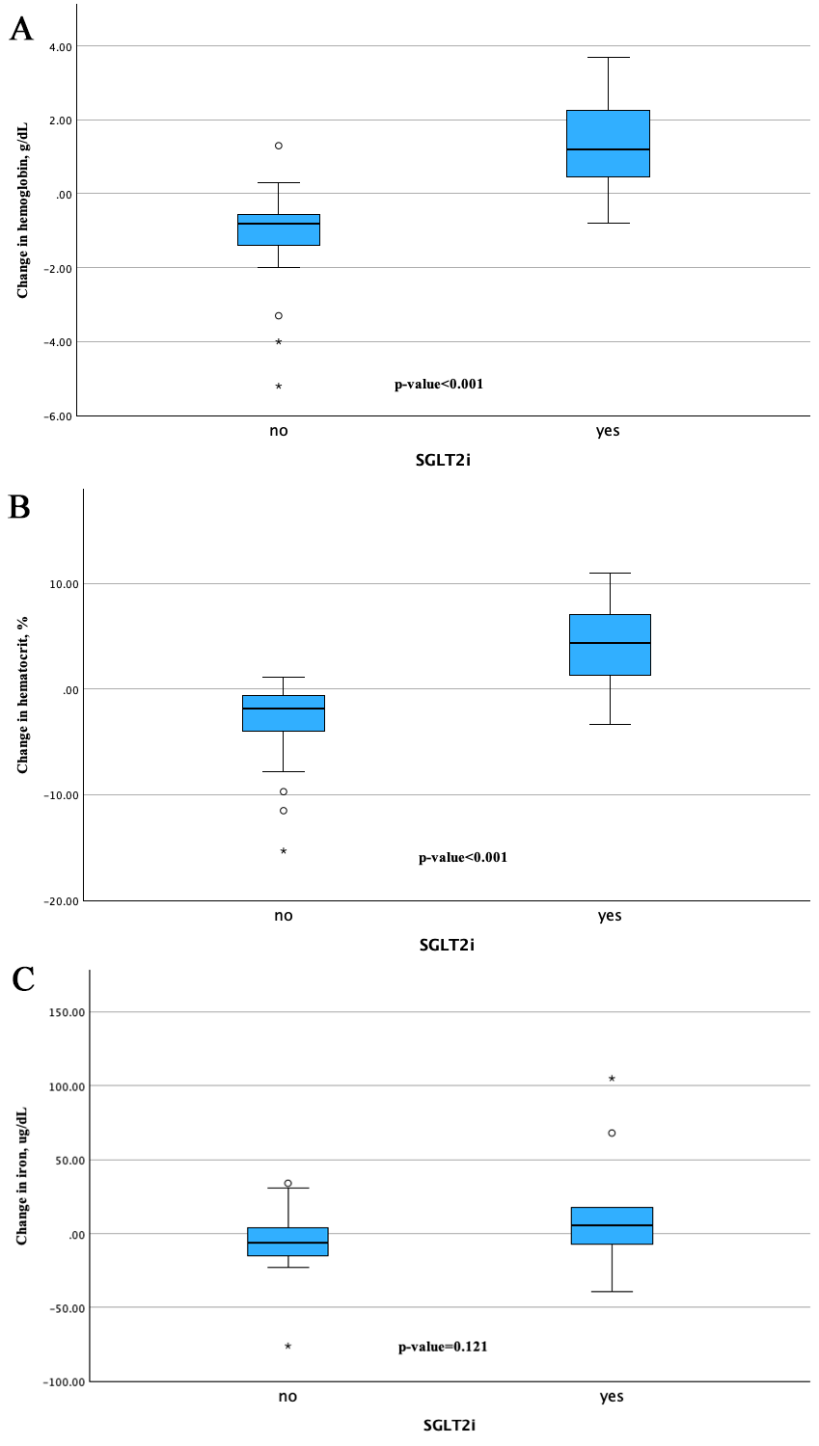
**Change from baseline in hemoglobin, hematocrit, and iron from baseline to follow-up assessment between the groups**. The changes in (A) hemoglobin and (B) hematocrit from baseline were significantly greater in the SGLT2i group compared to the control group. The change in (C) iron was also significant. “*” denotes outliers defined as values falling more than 1.5 times the IQR above the third quartile or below the first quartile. “circle” denotes outliers defined as values outside the IQR, but within 1.5 times the IQR from the third or the first quartile. IQR, interquartile rage; SGLT2i, sodium–glucose cotransporter 2 inhibitors.

The analysis of the laboratory parameters between the two groups showed a statistically significant increase in the mean hemoglobin level as well as in the mean hematocrit level in the SGLT2i group. Hemoglobin levels increased from 12.9 [12.6 to 13.1] to 14.2 [13.4 to 15.3] g/dL (*p*-value < 0.001), and hematocrit levels increased from 39.2 [37.4 to 39.6] to 42.9 [40.7 to 46.5] % (*p*-value < 0.001). In comparison, the participants without SGLT2i treatment demonstrated a significant decrease in both parameters, with hemoglobin levels decreasing from 12.5 [11.7 to 13.1] to 11.6 [10.5 to 12.2] g/dL (*p*-value < 0.001) and hematocrit levels decreasing from 36.8 [35.5 to 38.1] to 34.7 [33.0 to 36.1] % (*p*-value < 0.001), as illustrated in Fig. 1A,B.

Regarding the difference in iron levels and their change from BL to FUP assessments, there were no statistically significance changes. The SGLT2i group showed an improvement in the iron level from 70.5 [61.0 to 78.8] to 76.0 [66.0 to 107.5] µg/dL, while the control group demonstrated a slight decrease from 61.0 [48.0 to 76.5] to 60.0 [36.0 to 74.5] µg/dL, with *p*-value of 0.121 as shown in Fig. 1C.

In addition to the abovementioned laboratory parameters, we observed changes in NT-proBNP levels between patients treated with SGLT2i and those in the control group. Patients receiving SGLT2i therapy showed a decrease in NT-proBNP from BL to FUP, with a median change of –299.0 pg/mL [–1085.0 to 121.0], whereas the control group showed an increase of 117.0 pg/mL [167.0 to 5224.0]. However, this difference was not statistically significant (*p*-value = 0.149). Similarly, no significant improvements or declines in eGFR levels were observed between the two groups. Patients treated with SGLT2i exhibited a median change of –1.7 mL/min/1.73 m^2^ [–4.3 to 2.4], compared to –0.2 mL/min/1.73 m^2^ [–4.5 to 3.4] in the control group (*p*-value = 0.499). There was no significant change in ejection fraction during follow-up as can be seen in **Supplementary Table 1**. Univariate linear regression analysis for changes in laboratory parameters is represented in **Supplementary Table 2**. Other parameters are detailed in Table 4.

**Table 4.** **Differences in the laboratory parameters between the groups from baseline to follow-up**.

Variables	All (n = 40)	Patients without SGLT2i (n = 20)	Patients with SGLT2i (n = 20)	*p*-value
Hemoglobin, g/dL	0.0 (–0.8 to 1.3)	–0.8 (–1.2 to –0.6)	1.2 (0.5 to 2.2)	<0.001
Hematocrit, %	0.8 (–1.9 to 4.3)	–1.8 (–3.7 to –0.7)	4.4 (1.4 to 6.8)	<0.001
MCV, fL	–0.1 (–2.1 to 2.7)	0.6 (–1.7 to 2.8)	–0.3 (–2.5 to 1.6)	0.330
MCHC, g/dL	–0.3 (–0.8 to 0.1)	–0.6 (–0.9 to –0.2)	0.0 (–0.8 to 0.3)	0.070
RDW, %	0.5 (–0.2 to 1.0)	0.4 (0.0 to 1.1)	0.5 (–0.5 to 0.9)	0.935
Iron blood level, µg/dL	–3.0 (–12 to 11)	–6.0 (–15.0 to 4.0)	5.5 (–5.0 to 17.5)	0.121
Serum ferritin, µg/dL	–35.0 (–63.2 to 32.8)	–19.9 (–63.0 to 26.3)	–41.8 (–76.3 to 28.7)	0.793
TSAT, %	–0.8 (–2.9 to 7.5)	–2.1 (–3.0 to 0.4)	1.6 (–1.9 to 8.4)	0.138
NT-proBNP, pg/mL	–221.0 (703.0 to 806.0)	117.0 (1670.0 to 5224.0)	–299.0 (–1085.0 to 121.0)	0.149
eGFR, mL/min/1.73 m^2^	–0.3 (–4.5 to 3.0)	–0.2 (–4.5 to 3.4)	–1.7 (–4.3 to 2.4)	0.499

Values as a median and interquartile rage [IQR]. eGFR, estimated glomerular filtration rate; MCHC, mean corpuscular hemoglobin concentration; MCV, mean corpuscular volume; NT-proBNP, N-terminal prohormone of brain natriuretic peptide; RDW, red cell distribution width; TSAT, transferrin saturation; SGLT2i, sodium–glucose cotransporter 2 inhibitors.

### 3.3 Changes in CPET Parameters

CPET assessments revealed a stability of the parameters within the SGLT2i group compared to a decline observed in the control group, in the primary parameters of peak VO_2_ and the VE/VCO_2_ slope. Peak VO_2_, a crucial indicator of aerobic and cardiovascular capacity, exhibited a more favorable trend in the SGLT2i group, which showed a slight improvement of 0.1 [–1.4 to 0.8] compared to a decline in the control group of –1.3 [–2.3 to –0.2], though this difference did not reach statistical significance (*p* = 0.206). The VE/VCO_2_ slope, an indicator of ventilatory efficiency and an important prognostic marker in heart failure, increased slightly in the control group 2.6 [–1.6 to 8.9] while remaining essentially unchanged in the SGLT2i group 0.1 [–1.1 to 5.8] with a *p*-value of 0.964. Additional CPET parameters are presented in Table 5. Univariate linear regression analysis for changes in CPET parameters can be seen in **Supplementary Table 3**.

**Table 5.** **Differences in the cardiopulmonary exercise testing parameters between the groups from baseline to follow-up**.

Variables	All (n = 40)	Patients without SGLT2i (n = 20)	Patients with SGLT2i (n = 20)	*p*-value
Peak VO_2_, mL/min	–67 (–120 to 51)	–67 (–119 to –10)	–65 (–149 to 58)	0.810
Peak VO_2_, mL/min/kg	–0.7 (–1.7 to 0.6)	–1.3 (–2.3 to –0.2)	0.1 (–1.4 to 0.8)	0.206
VO_2_ at AT, mL/min	–0.2 (–1.3 to 0.8)	–0.5 (–1.2 to 0.1)	0.5 (–1.2 to 1.1)	0.213
VE/VCO_2_ slope	2.2 (–1.2 to 8.4)	2.6 (–1.6 to 8.9)	0.1 (–1.1 to 5.8)	0.964
Peak VE, L/min	–1.0 (–3.0 to 6.0)	3.0 (–1.0 to 10.0)	–2.0 (–5.2 to –1.0)	0.063
Peak workload, Watt	–3.0 (–40.0 to 20.0)	–5.5 (–13.8 to 2.8)	–0.4 (–6.5 to 5.8)	0.507
Peak RER	0.0 (0.0 to 0.1)	0.1 (0.0 to 0.1)	0.0 (0.0 to 0.0)	0.701
Peak HR, bpm	0.3 (–10.0 to 10.1)	–6.0 (–18.0 to 6.1)	7.1 (–10.9 to 25.0)	0.221

Values a median and interquartile rage [IQR]. AT, anaerobic threshold; HR, heart ratio; RER, respiratory exchange ratio; VE, pulmonary ventilation; VO_2_, oxygen consumption; SGLT2i, sodium–glucose cotransporter 2 inhibitors; VCO_2_, carbon dioxide production.

**Fig. 2.**
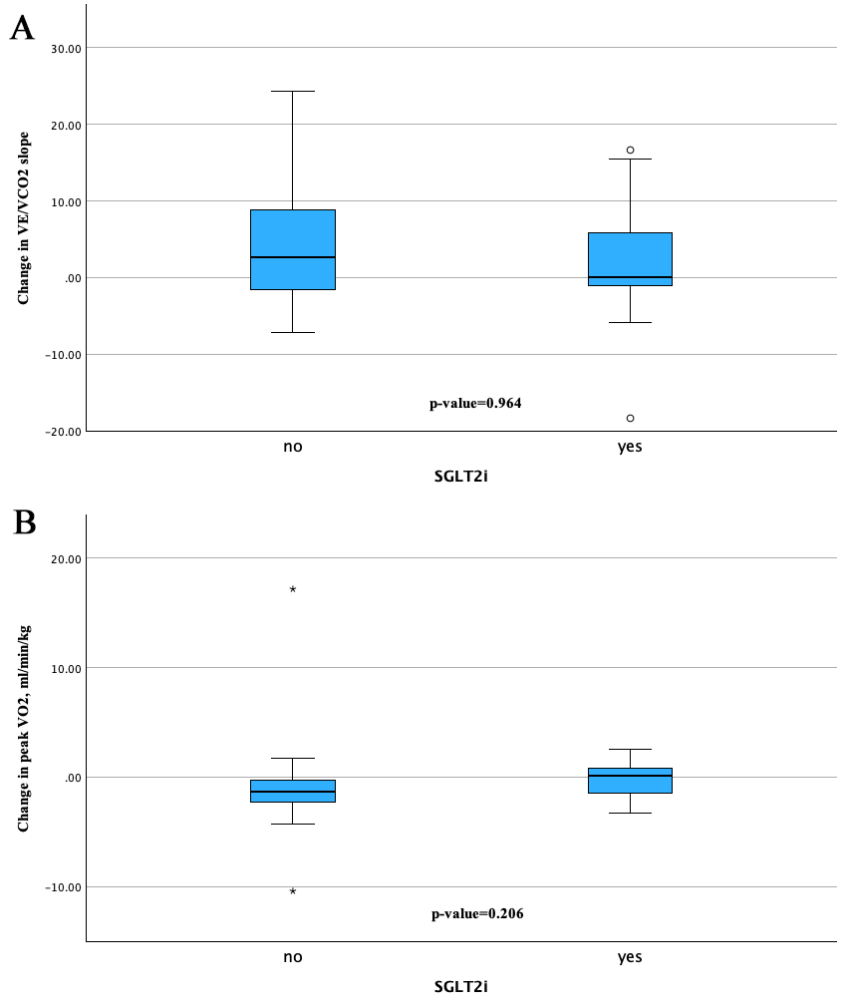
**Change from baseline in slope and peak VO_2_**. (A) describes changes over time in VE/VCO_2_ slope between the SGLT2i group and the control group and (B) provides the changes in peak VO_2_ from baseline to follow-up. “*” denotes outliers defined as values falling more than 1.5 times the IQR above the third quartile or below the first quartile. “circle” denotes outliers defined as values outside the IQR, but within 1.5 times the IQR from the third or the first quartile. IQR, interquartile rage; VE, pulmonary ventilation; VCO_2_, carbon dioxide production; SGLT2i, sodium–glucose cotransporter 2 inhibitors.

As illustrated in Fig. 2A,B, the contrast in VE/VCO₂ slope and peak VO_2_ trends suggest that patients in the SGLT2i group were stable throughout the study period, while those in the control group experienced a slight decline.

## 4. Discussion

This study provides new insights into the hematopoietic effects of SGLT2i in patients with CA. CA patient who received treatment with SGLT2i in addition to BMT, had a significant improvement in hemoglobin and hematocrit levels and had a stable FC in comparison to the control group which did not receive SGLT2i. In addition, there was a reduction in cardiac biomarker level in the SGLT2i group. This is the first study to demonstrate the hematopoietic effect of SGLT2i as well as their effect on FC in CA patients.

The hematopoietic benefits of SGLT2i from the CANDLE trial have been documented in patients with HF and diabetes [38], however there is still limited evidence regarding these benefits in patients with CA. Ghanim *et al*. [39] described a suppressive effect of dapagliflozin on hepcidin, a known suppressor of erythropoiesis, in patients with diabetes mellitus. Along with diabetes, amyloid fibrils are known for their toxic effect on cells and different organs of the body [3, 40], that can cause chronic inflammation and could be reflected in excessive production of hepcidin and ferritin. Hepcidin serum concentration was not collected in our study. However, we observed a more significant decrease in ferritin level in our SGLT2i group compared to the control group during follow-up (Table 4) which might reflect comparable effects of erythropoiesis suppression in CA patients. Another mechanism that was mentioned by Steinhardt *et al*. [27], may be associated with the osmotic diuretic effect of SGLT2i, leading to increased excretion of free water. With significant improvements in hemoglobin and hematocrit levels, and positive trends of iron and TSAT levels in the SGLT2i group, this hypothesis might also be applicable in our patients (Table 4, Fig. 1C).

Previous studies have demonstrated the importance of assessing FC using CPET in patients with CA [14, 41, 42]. Patients with lower peak VO_2_ are known to have a poorer prognosis and different cut-off values have been proposed for predicting patient outcomes [14, 41, 43]. In our study, the historical control group and SGLT2i treated group had a peak VO_2_ of 13 and 14 mL/kg/min at BL, respectively. Moreover, these cut-off values for peak VO_2_ are particularly relevant for anemia, as anemia can significantly impair oxygen delivery to tissues, further reducing peak VO_2_ and making it more challenging for patients to perform physical activities [44]. This might imply a potentially worse prognosis in patients without SGLT2i, as they reach both proposed cut-offs for peak VO_2_. Furthermore, the group without SGLT2i treatment demonstrated a worse VO_2_ at AT during BL assessment, implying a more limited cardiac output in that group. Cardiac output is driven by both heart rate and stroke volume. Since patients with CA tend to have a rather fixed stroke volume, the group without SGLT2i might show more advanced disease, associated with the disease pathophysiology of CA [45, 46].

CPET revealed a decline in FC in the control group, evidenced by a reduction of >1 mL/kg/min of peak VO_2_ during follow-up [14], in contrast with the stability in the SGLT2i group. The decrease in peak VO_2_ could indicate disease progression during follow-up. In patients receiving SGLT2i therapy in addition to BMT a positive effect on limiting disease progression could be demonstrated in our study.

One of the key biomarkers used to assess cardiac stress and monitor the disease progression is NT-proBNP [8, 47]. In our study, we observed a decrease in NT-proBNP levels in the SGLT2i-treated group compared to the control group. This reduction may be attributed to the direct cardioprotective effects of SGLT2i, which include mechanisms beyond their initial glucose-lowering properties. SGLT2 inhibitors may exert beneficial effects on myocardial function by reducing oxidative stress, enhancing mitochondrial efficiency, and attenuating adverse cardiac remodeling [48].

SGLT2i has been shown to improve myocardial energy efficiency by shifting the substrate metabolism from glucose to ketone bodies, which are a more efficient fuel source for the heart, especially in conditions of increased metabolic stress, such as CA. This metabolic shift not only enhances cardiac energy production but also helps stabilize cardiomyocyte function and reduce left ventricular mass, thus mitigating the progressive myocardial stiffening often observed in CA [49].

In contrast, the increase in NT-proBNP levels observed in the control group aligns with the natural course of CA, where continued amyloid deposition and resultant cardiac remodeling exacerbate HF symptoms and increase myocardial stress. This disparity in NT-proBNP trends between the SGLT2i-treated and control groups may imply that SGLT2i provides a protective effect against the worsening of cardiac function typically seen in CA, potentially leading to improved clinical outcomes and better prognosis.

In general, patients with CA are elderly and suffer from multi-organ manifestation, such as atrial fibrillation, aortic stenosis and renal dysfunction [6, 50]. It should be noted that patients without SGLT2i therapy were, on average, older at study entry, which could have been a contributing factor to the development and progression of anemia in our patients [51]. Another contributing factor that was observed in our study, is the dominant prevalence of chronic kidney disease (CKD) at BL assessment in the control group compared to the SGLT2i group (60 % vs. 30%, respectively). There is known strong correlation between advanced CKD and the likelihood of developing anemia [52]. These differences in age and CKD between the groups might have influenced the observed changes in hemoglobin and hematocrit levels.

CA is strongly associated with the prevalence of CKD and adversely affects kidney function [53]. This relationship further supports a higher incidence of CKD observed in both groups. Additionally, we noted a slight decrease in eGFR levels in treatment group from BL to FUP visit. Our findings are in line with those previously reported in the literature [27, 29]. In contrast, Porcari *et al*. [26] observed a sustained slower rate of decline in eGFR over the long-term FUP in CA patients. The importance of long-term monitoring to capture the full spectrum of renal effects associated with SGLT2i is essential and should be implemented in further studies using SGLT2i in amyloidosis patients.

## 5. Limitations

Several limitations of the present study need to be acknowledged. First, it was a single-center study, which may limit the generalizability of the results to broader populations. The specific characteristics of the single center may introduce biases that are not representative of other clinical settings.

Second, the sample size was limited, particularly due to the relatively rare occurrence of CA.

Third, due to a lack of awareness regarding the use of SGLT2i therapy for treatment of all subtypes of HF, some patients were not eligible for follow-up assessments due to discontinuation of SGLT2i therapy mediated by primary care physician.

Furthermore, the study lacks detailed information on whether the patients received iron supplementations during the study period. This is a significant limitation, as iron supplementation could have a substantial impact on the hematological parameters, such as hemoglobin, hematocrit, and iron levels, which were key outcomes of this study. In our clinical setting, no patients received iron supplementation as part of their treatment protocol. Furthermore, all patients were regularly questioned regarding any external iron supplementation, and none reported taking iron supplements outside our clinic. Despite these measures, we cannot completely exclude the possibility of unreported supplementation, which could introduce a potential interaction.

## 6. Conclusions

In summary, our study demonstrated the beneficial effects of SGLT2i on hematological parameters, exercise performance, and cardiac biomarker in patients with CA. Patients treated with SGLT2i therapy showed stability or improvements compared to the control group. Despite the study limitations, our findings suggest that SGLT2i could play a valuable role in managing CA. Further multi-center studies with large cohorts and consistent FUP are warranted to validate these results.

## Data Availability

The data underlying this article will be shared on reasonable request to the corresponding author.

## Abbreviations

6MWT, 6-minutes walking test; AL, immunoglobulin light chain amyloid; AT, anaerobic threshold; ATTR, transthyretin amyloid; ATTRv, variant transthyretin amyloid; ATTRwt, wild-type transthyretin amyloid; BL, baseline assessment; BMT, best medical therapy; CA, cardiac amyloidosis; CKD, chronic kidney disease; CM, cardiomyopathy; CPET, cardiopulmonary exercise testing; eGFR, estimated glomerular filtration rate; FC, functional capacity; FUP, follow-up assessment; HF, heart failure; HFpEF, heart failure with preserved ejection fraction; LVEF, left ventricle ejection fraction; MCHC, mean corpuscular hemoglobin concentration; MCV, mean corpuscular volume; NT-proBNP, N-terminal prohormone of brain natriuretic peptide; NYHA, New York Heart Association; peak VE, peak pulmonary ventilation; peak VO_2_, peak oxygen consumption; RDW, red cell distribution width; sFLC, serum free light chain; SGLT2i, sodium–glucose cotransporter 2 inhibitors; TSAT, transferrin saturation; TTE, transthoracic echocardiography; VCO_2_, carbon dioxide production; VE, pulmonary ventilation; VO_2_, oxygen consumption.

## Supplementary Material

Supplementary material associated with this article can be found, in the online version, at https://doi.org/10.31083/RCM26081.
