# Supplementary material for: Potential Hematopoietic Effects of SGLT2 Inhibitors in Patients with Cardiac Amyloidosis
Source: Rev Cardiovasc Med. 2025 Mar 20;26(3):26081. doi: 10.31083/RCM26081 (PMC11951278; doi:10.31083/RCM26081)
Supplement: Supplementary file 1 [file 2153-8174-26-3-26081-s1.docx]

**Supplemental Table 1**. Changes in ejection fraction between two study groups.

| Variables | All (n=40) | Patients without SGLT2  (n=20) | Patients with SGLT2 (n=20) | p-value |
| --- | --- | --- | --- | --- |
| HFrEF/HFmrEF/HFpEF,%  Baseline | (17.5;17.5;60.0) | (20.0;30.0;50.0) | (15.0;5.0;70.0) | 0.095 |
| HFrEF/HFmrEF/HFpEF,%  Follow-up | (20.0;25.0;55.0) | (25.0;30.0;45.0) | (15.0;20.0;65.0) | 0.377 |

Supplemental Table 1 shows changes over time in the ejection fraction. Values as N (%). HFmrEF, heart failure with mild reduced ejection fraction; HFpEF, heart failure with preserved ejection fraction; HFrEF, heart failure with reduced ejection fraction.

**Supplemental Table 2.** Univariate linear regression for laboratory data.

|  |  | Estimate (CI) | p-value |
| --- | --- | --- | --- |
| Hemoglobin, g/dL | Follow-up | 2.899 (1.9,3.9) | < 0.001 |
|  | Baseline | 0.01 (-0.61,0.63) | 0,973 |
| Hematocrit, % | Follow-up | 8.72 (5.73,11.71) | < 0.001 |
|  | Baseline | -0.056 (-0.65,0.54) | 0,849 |
| MCV, fL | Follow-up | 0.069 (-2.36,2.5) | 0,954 |
|  | Baseline | -0.287 (-0.52,-0.06) | 0,015 |
| MCHC, g/dL | Follow-up | 0.173 (-0.38,0.72) | 0,528 |
|  | Baseline | -0.273 (-0.51,-0.03) | 0,027 |
| RDW, % | Follow-up | -0.311 (-1.27,0.65) | 0,515 |
|  | Baseline | -0.59 (-0.79,-0.39) | < 0.001 |
| Iron blood level, ug/dL | Follow-up | 26.591 (7.31,45.87) | 0,009 |
|  | Baseline | -0.787 (-1.23,-0.34) | 0,001 |
| Serum Ferritin, ug/dL | Follow-up | 27.566 (-66.19,121.32) | 0,548 |
|  | Baseline | -0.503 (-0.74,-0.27) | < 0.001 |
| TSAT, % | Follow-up | 8.122 (0.83,15.42) | 0,031 |
|  | Baseline | -0.666 (-1.19,-0.14) | 0,016 |
| NT-proBNP, pg/mL | Follow-up | -1154.433 (-2189.76,-119.11) | 0,03 |
|  | Baseline | -0.186 (-0.41,0.03) | 0,093 |
| eGFR, mL/min/1.73 m^2^ | Follow-up | -1.107 (-6.51,4.3) | 0,68 |
|  | Baseline | -0.101 (-0.21,0.01) | 0,076 |

Supplemental Table 2 shows linear regression analysis of changes in laboratory data. Estimates and p-values for the effects on changes of laboratory parameters from a model including SGLT2i and covariates baseline, age and gender are presented; age and gender were never significant and are therefore omitted. eGFR, estimated glomerular filtration rate; MCHC, mean corpuscular hemoglobin concentration; MCV, mean corpuscular volume; NT-proBNP, N-terminal prohormone of brain natriuretic peptide; RDW, red cell distribution width; TSAT, transferrin saturation.

**Supplemental Table 3.** Univariate linear regression for CPET data.

|  |  | Estimate (CI) | p-value |
| --- | --- | --- | --- |
| Peak VO_2_, mL/min | Follow-up | -11.527 (-185.16,162.1) | 0,892 |
|  | Baseline | 0.087 (-0.15,0.32) | 0,452 |
| Peak VO_2_, ml/min/kg | Follow-up | 1.559 (-2.03,5.15) | 0,378 |
|  | Baseline | -0.269 (-0.69,0.16) | 0,205 |
| VO_2_ at AT, mL/min | Follow-up | 2.078 (0.48,3.68) | 0,013 |
|  | Baseline | -0.733 (-1.16,-0.31) | 0,002 |
| VE/VCO_2_ slope | Follow-up | -1.465 (-9.21,6.28) | 0,699 |
|  | Baseline | 0.296 (-0.14,0.74) | 0,177 |

Supplemental Table 3 shows linear regression analysis of changes in CPET data. Estimates and p-values for the effects on changes of CPET parameters from a model including SGLT2i and covariates baseline, age and gender are presented; age and gender were never significant and are therefore omitted. AT, anaerobic threshold; VE, minute ventilation; VO_2_, oxygen consumption.
